# Preference for Resistance Training Volume After Acute Exercise in Women With Fibromyalgia: A Randomized Crossover Clinical Trial

**DOI:** 10.1002/pri.70364

**Published:** 2026-10-16

**Authors:** Bruno Honório Cavalcanti, André Pontes‐Silva, Gleidson Mendes Rebouças, Areolino Pena Matos, Clécio Gabriel de Souza, Mariana Arias Avila, Marcelo Cardoso de Souza

**Affiliations:** ^1^ Postgraduate Program in Physical Therapy Faculty of Health Sciences of Trairi Federal University of Rio Grande do Norte Santa Cruz RN Brazil; ^2^ Postgraduate Program in Physical Education NERD Research Group Federal University of Maranhão São Luís MA Brazil; ^3^ School of Physical Education Exercise Neurophysiology Laboratory (LaNEx) University of the State of Rio Grande do Norte Mossoró RN Brazil; ^4^ Faculty of Physical Therapy and Occupational Therapy Postgraduate Program in Human Movement Sciences Federal University of Pará Belém PA Brazil; ^5^ Postgraduate Program in Physical Therapy, Department of Physical Therapy Federal University of São Carlos São Carlos Brazil; ^6^ Department of Physical Therapy Federal University of Rio Grande do Norte Natal RN Brazil

**Keywords:** exercise, fibromyalgia, pain, resistance training

## Abstract

**Background and Purpose:**

To evaluate participants' preferences following acute exposure to three resistance training protocols differing solely in the number of sets performed.

**Methods:**

Randomized crossover clinical trial. Thirty‐eight women aged 18–65 years with fibromyalgia diagnosed according to the 2016 American College of Rheumatology criteria completed the study. All participants were naïve to resistance training during the previous 6 months and completed every intervention protocol and assessment. Participants performed three resistance training sessions in randomized order, consisting of one‐, two‐, or three‐set protocols separated by 7‐day washout periods. Exercise selection, intensity, and repetitions were standardized across protocols, with the number of sets representing the only intervention variable. The primary outcome was participant preference for resistance training volume after completion of all three protocols. Secondary outcomes included treatment expectancy, pain intensity assessed before and after exercise and 24 hours later, and exercise execution time.

**Results:**

No participant selected the one‐set protocol as the preferred training volume, whereas the two‐ and three‐set protocols were equally preferred, each being selected by 50% of participants (*p* < 0.001; Cohen's *w* = 0.71). Treatment expectancy did not differ significantly among protocols (*p* = 0.20). No between‐protocol differences in pain intensity were observed at any assessment time point. A significant within‐protocol reduction in pain over time was identified only for the two‐set protocol (*p* = 0.015); however, the magnitude of change did not reach the predefined minimal clinically important difference. Exercise execution time did not differ significantly among training volumes, and no serious adverse events occurred during the study. Discussion: Women with fibromyalgia demonstrated equivalent preference for resistance training protocols involving two and three sets, whereas no participant identified the one‐set protocol as the preferred option. Treatment expectancy was similar across protocols, and no clinically meaningful differences in pain intensity were observed. These findings support the incorporation of patient preference into individualized resistance training prescriptions and warrant further investigation of whether such preferences translate into improved long‐term adherence and clinical outcomes.

**Trial Registration:**

ClinicalTrials.gov (Identifier: NCT06424743, 2024‐08‐21)

## 1 Introduction

Fibromyalgia is a chronic pain condition characterized by widespread musculoskeletal pain and a constellation of associated symptoms, including fatigue, sleep disturbances, and cognitive and mood alterations. It affects a substantial proportion of the global population and represents a significant clinical and socioeconomic burden. Although pain is the defining feature of the syndrome, its impact extends beyond nociception, influencing physical functioning, emotional well‐being, and quality of life (Häuser et al. 2015).

Advances in the understanding of fibromyalgia have highlighted its complex and multifactorial pathophysiology (González‐Álvarez, Riquelme‐Aguado, Rossettini, et al. 2024). Alterations in central pain processing, dysregulation of neuroendocrine and autonomic systems, and neuroinflammatory mechanisms contribute to symptom persistence and heterogeneity (Häuser et al. 2015). This multidimensional profile supports the use of therapeutic strategies that move beyond symptom suppression and address biological, psychological, and behavioral dimensions of the condition. In this context, non‐pharmacological interventions—particularly physical exercise—have assumed a central role in fibromyalgia management (Macfarlane et al. 2017).

Resistance training has emerged as an effective intervention for individuals with fibromyalgia (Pontes‐Silva et al. 2025a; Pontes‐Silva et al. 2025b), with evidence supporting improvements in pain, physical function, muscular strength, and overall quality of life (Macfarlane et al. 2017). Despite its established benefits, resistance training prescription in fibromyalgia remains challenging due to the absence of precise recommendations regarding acute training variables (Schoenfeld et al. 2017). Parameters such as intensity, exercise selection, execution tempo, and training volume are often extrapolated from non‐clinical populations, despite the unique physiological and perceptual characteristics of this group (Schoenfeld et al. 2017).

Among these variables, training volume—commonly operationalized as the product of sets, repetitions, and load—has been identified as a major determinant of training‐induced adaptations (Schoenfeld et al. 2017). From a conceptual standpoint, training volume can be viewed as analogous to a therapeutic dose, whereby variations in the prescribed amount may elicit distinct physiological and perceptual responses (Forner‐Álvarez et al. 2024; Schoenfeld et al. 2017). This analogy is particularly relevant in fibromyalgia, where tolerance to physical stress is frequently reduced and symptom exacerbation may occur when exercise demands exceed individual capacity (Forner‐Álvarez et al. 2024).

Muscle‐related impairments have been documented in individuals with fibromyalgia (Melo et al. 2026), including mitochondrial dysfunction, reduced oxidative capacity, and altered muscle metabolism (González‐Álvarez, Riquelme‐Aguado, González‐Pérez, et al. 2024). These alterations may contribute to early fatigue, pain amplification, and reduced exercise tolerance (Vancampfort et al. 2024). Consequently, inappropriate resistance training dosing may not only limit therapeutic benefits but also increase the likelihood of negative exercise experiences, reinforcing avoidance behaviors and disengagement from physical activity (Pontes‐Silva et al. 2023).

Exercise adherence remains a critical concern in fibromyalgia care (Andrade et al. 2020). Many individuals report fear of symptom worsening, heightened pain during or after exercise, and low confidence in their ability to tolerate training demands. Evidence suggests that even when resistance training programs are clinically effective and safe, these attributes alone are insufficient to guarantee long‐term adherence (Pontes‐Silva et al. 2025; Pontes‐Silva et al. 2025). Psychological and experiential factors play a decisive role in whether patients continue or abandon exercise interventions (Vancampfort et al. 2024).

Within the framework of evidence‐based practice, patient preference has long been recognized as a key component in clinical decision‐making (Andrade et al. 2018). In fibromyalgia specifically, incorporating individual preferences into exercise prescription has been recommended to enhance engagement, acceptability, and sustainability of interventions. However, despite growing emphasis on personalized exercise approaches, limited attention has been given to how specific resistance training variables—such as training volume—align with patient preferences (Viceconti et al. 2021).

Although previous studies have demonstrated that a wide range of resistance training volumes can be beneficial for individuals with fibromyalgia (Pontes‐Silva et al. 2025a; Pontes‐Silva et al. 2025b), investigations explicitly examining patient preference for different training volumes are lacking. Addressing this gap is essential to inform patient‐centered exercise prescription and to optimize both adherence and clinical relevance. Therefore, under the hypothesis that women with fibromyalgia may exhibit distinct preferences across resistance training volumes, this study aimed to evaluate participants' preferences following acute exposure to three resistance training protocols differing solely in the number of sets performed.

## 2 Methods

### 2.1 Trial Design and Setting and Ethics Aspects

This study was conducted as a randomized crossover clinical trial, previously registered at ClinicalTrials.gov (Identifier: NCT06424743; registration date: August 21, 2024) and approved by the Research Ethics Committee of the Federal University of Rio Grande do Norte (approval number: 6.303.419). The trial followed the recommendations of the SPIRIT Statement and was reported in accordance with the Consolidated Standards of Reporting Trials (CONSORT). The exercise intervention was described following the Consensus on Exercise Reporting Template (CERT) (Slade et al. 2016). The full study protocol, including detailed descriptions of the trial rationale, design, and planned procedures, has been published elsewhere (Doi: 10.1002/pri.70101) and should be consulted for additional methodological details (Cavalcanti et al. 2025).

### 2.2 Patient, Public Involvement, and Recruitment

Participants were recruited through advertisements disseminated via social media platforms. Initial contact was conducted by a trained researcher via telephone to schedule eligibility screening. Women who met the inclusion criteria were informed about the study objectives and procedures and provided written informed consent prior to participation. Sociodemographic information and data related to fibromyalgia impact were collected at baseline. To preserve blinding, participants were identified using numerical codes, and personal data were stored in a secure database.

### 2.3 Eligibility Criteria

Women aged 18–65 years with a diagnosis of fibromyalgia according to the 2016 American College of Rheumatology criteria were eligible for inclusion (Wolfe et al. 2016). The upper age limit of 65 years was prespecified in the study protocol and was selected based on the age range commonly adopted in exercise studies involving individuals with fibromyalgia, while aiming to reduce the potential influence of age‐related comorbidities and clinical heterogeneity on exercise responses.

### 2.4 Research Team and Safety Considerations

The study was conducted by a multidisciplinary research team with predefined roles: (A) participant screening and blinded assessments; (B) randomization and allocation procedures; (C) supervision of training protocols; (D) data tabulation; and (E) statistical analysis. No serious adverse events were reported during the study (Cavalcanti et al. 2025).

Participants were monitored throughout each training session by the supervising physiotherapists, and any adverse events or unexpected symptoms reported during or immediately after exercise were documented. No serious adverse events requiring medical intervention or discontinuation of participation occurred during the study.

### 2.5 Randomization, Allocation, Blinding, Patient Timeline, and Data Collection Methods

Randomization was performed prior to each training session to determine the order in which participants completed the three resistance training protocols (one, two, or three sets). Allocation was conducted using opaque, sealed, sequentially numbered envelopes, each containing one of the three protocol options. After completing a protocol, the corresponding option was removed before the subsequent draw, ensuring that all participants completed each protocol once, serving as their own controls. The order in which the three protocols were completed constituted the intervention sequence for each participant.

Allocation procedures were conducted by a researcher not involved in assessments or interventions. Outcome assessments were performed by a blinded evaluator who did not participate in training supervision. Due to the nature of the intervention, participants and therapists were not blinded. Participants were instructed not to disclose protocol details to other participants or researchers.

Following completion of the third training protocol, participants were contacted 1 week later to report their preferred training protocol. Data were subsequently tabulated and forwarded to an independent statistician who was blinded to previous study procedures.

### 2.6 Intervention and Comparator

All training sessions were conducted after publication of the study protocol. Prior to the first intervention, participants underwent a baseline assessment that included anthropometric measurements, clinical characterization, and familiarization with the exercises. Submaximal repetition testing was performed for the following exercises: barbell bench press, front lat pulldown, 45° leg press, and seated leg curl. These data were used to prescribe individualized training loads.

Each participant completed three resistance‐training sessions, differing only in the number of sets performed (one, two, or three sets). The four exercises were performed in a fixed order: barbell bench press, front lat pulldown, 45° leg press, and seated leg curl. For all protocols, participants performed 10 repetitions per exercise at approximately 60% of the estimated one‐repetition maximum (1RM), with 2 min of rest between sets. Thus, the one‐, two‐, and three‐set protocols comprised one, two, and three sets per exercise, respectively. The three sessions were separated by 7‐day washout periods (Table 1). Before and after each session, pain intensity and affect were assessed. Subjective perception of effort and total training volume were recorded after each session. The exercise protocol is summarized in Table 2. No modifications or deviations were made to the prespecified resistance‐training intervention. All participants completed the three training protocols as planned (Cavalcanti et al. 2025).

**TABLE 1:** Resistance‐training protocol.

Variable	One‐set protocol	Two‐set protocol	Three‐set protocol
Exercises	Bench press; front lat pulldown; 45° leg press; seated leg curl	Same	Same
Exercise order	1–4	1–4	1–4
Sets per exercise	1	2	3
Repetitions per set	10	10	10
Load	∼60% estimated 1RM	∼60% estimated 1RM	∼60% estimated 1RM
Rest between sets	2 min	2 min	2 min
Total sets/session	4	8	12
Washout	7 days	7 days	7 days

### 2.7 Assessments and Baseline Characterization

Baseline assessments were conducted to characterize the sample and establish reference values. Data collected included age, educational level, anthropometric measures, time since diagnosis, physical activity level, treatment expectancy, and fibromyalgia impact. These characteristics are fully described in the published protocol and are not reproduced here to avoid redundancy (Cavalcanti et al. 2025).

### 2.8 Load Determination

Training loads were determined using a validated submaximal repetition test to estimate the one‐repetition maximum, minimizing injury risk and enhancing clinical applicability. The prediction equation used was: 1RM = load × [1 + (0.025 × repetitions)] (O’Shea et al. 1989). All tests were preceded by a standardized warm‐up consisting of 5–10 repetitions at 40%–60% of perceived maximal load, followed by a two‐minute rest period. Load testing was performed during the baseline assessment using standardized resistance machines.

For machine‐based exercises, only the externally added weight plates were recorded in the database. The intrinsic resistance of each machine was not included in these values, meaning that the reported loads should be interpreted as additional external loads rather than the total resistance experienced by participants.

**TABLE 2:** Pain intensity across assessment time points for each resistance training volume (*n* = 38).

Assessment time point	One set	Two sets	Three sets	Between‐protocol *p* value
Baseline	4.97 ± 1.92	4.97 ± 1.92	4.97 ± 1.92	
Pre‐exercise	4.53 ± 2.47	4.55 ± 2.23	4.47 ± 2.48	0.987
Post‐exercise	4.05 ± 2.25	3.76 ± 2.07	3.84 ± 2.48	0.797
24 h post‐exercise	4.39 ± 2.24	4.71 ± 1.89	4.32 ± 2.61	0.594
Within‐protocol time effect	*p* = 0.130	*p* = 0.015	*p* = 0.074	

*Note:* Values are presented as mean ± standard deviation. Baseline = 1 week before the first training session; Pre‐exercise = immediately before the training session; Post‐exercise = immediately after the training session; 24 h post‐exercise = 24 hours after the training session. Between‐protocol comparison: repeated‐measures ANOVA (training volume effect). Greenhouse–Geisser corrected *p* value (sphericity not assumed). For the two‐set protocol, post hoc pairwise comparisons identified significant differences between *baseline and post‐exercise* and between *pre‐exercise and post‐exercise* (*p* < 0.05). No other pairwise comparisons were statistically significant.

The number of repetitions recorded during the submaximal testing phase should not be interpreted as the target number of repetitions for the intervention sessions. During testing, participants performed as many repetitions as possible with a given load to allow estimation of 1RM. In contrast, during the intervention sessions, training loads were prescribed at approximately 60% of the estimated 1RM, and the number of repetitions was intentionally standardized at 10 for all participants and protocols, without requiring execution to concentric failure.

### 2.9 Outcomes

The primary outcome was participant preference for training volume, assessed after completion of all three protocols. Secondary outcomes included treatment expectancy, pain intensity, and exercise cadence. The timing and operational definitions of all outcomes are described in detail in the published protocol (Cavalcanti et al. 2025).

### 2.10 Expectation

Treatment expectancy was assessed using a Likert‐type scale designed to evaluate participants' expectations regarding the anticipated effects of each training protocol at baseline, prior to any intervention. Participants were asked to rate their expected response to resistance training performed with one, two, and three sets using a five‐point scale ranging from “get a lot worse” (1) to “get a lot better” (5), following previously established procedures. Expectancy was assessed separately for each training volume to minimize response bias and to ensure independent evaluation of anticipated outcomes across protocols (Beasley et al. 2017).

### 2.11 Pain Intensity

Widespread pain intensity was assessed using the Numeric Pain Rating Scale, a self‐report instrument validated for use in Portuguese‐speaking populations (Ferreira‐Valente et al. 2011). The scale ranges from 0 (“no pain”) to 10 (“the worst pain imaginable”). A reduction of at least two points was considered indicative of a minimal clinically important difference (Farrar et al. 2001). Pain intensity was recorded at three time points for each training session: immediately before the session, immediately after completion, and 24 hours post‐exercise.

### 2.12 Cadence

Exercise cadence was assessed as an objective indicator of physical effort during resistance training. The duration of each set, encompassing both concentric and eccentric phases, was measured using a stopwatch. Timing commenced at the initiation of the first repetition and ceased upon completion of the final repetition of each set. Recorded values were documented in a standardized spreadsheet and used to estimate effort time independently of subjective exertion ratings (Pontes‐Silva 2024).

### 2.13 Participant and Audience Engagement

Participants were not involved in the study design, formulation of the research question, or development of recruitment strategies. Upon completion of the study, participants were offered the opportunity to receive a summary of the study findings. If differential responses or preferences among training protocols were identified, these results were communicated to participants in an accessible format.

### 2.14 Sample Size

Sample size calculation was performed a priori using G*Power software (version 3.1.9.7) with the Chi‐squared test for comparisons between the proportions of self‐reported preferences observed in the contingency tables. We calculated the sample seeking an effect size of 0.8 in the difference in the primary outcome (preference). That is, w = 0.8, *α* = 0.05, *β* = 0.80, non‐centrality parameter *λ* = 23.04, and critical Chi‐squared test = 37.65 (Gignac and Szodorai 2016; Pontes‐Silva 2023). Thus, the sample was estimated to be 36 patients (Cavalcanti et al. 2025).

### 2.15 Statistical Methods

Data were analyzed using appropriate statistical software (SPSS, version 27). Initially, descriptive analyses were conducted for continuous variables using means and standard deviations, along with minimum and maximum values and their respective 95% confidence intervals (95% CIs). Categorical variables were summarized using absolute and relative frequencies, also reported with 95% CIs.

Data normality was assessed using the Kolmogorov–Smirnov test, while homogeneity of variances was evaluated using Levene's test. When applicable, sphericity was examined using Mauchly's test, and the Greenhouse–Geisser correction was applied when the sphericity assumption was violated.

Anthropometric, clinical, metabolic, and behavioral characteristics of the sample were presented descriptively, without inferential comparisons, as the study involved a single cohort. Sociodemographic variables, continuous medication use, and physical activity level classification (International Physical Activity Questionnaire [IPAQ]) were analyzed using contingency tables. Observed proportions were compared using the chi‐square test, with χ^2^ statistics and corresponding *p* values reported.

Preference for training volume (one, two, or three sets), defined as the primary outcome, was analyzed using the chi‐square test to compare observed frequencies across training protocols. Treatment expectancy was compared among the three training protocols using a one‐way analysis of variance (ANOVA), with the training protocol treated as a fixed factor.

Pain intensity was analyzed at four time points (baseline, pre‐exercise, post‐exercise, and 24 hours post‐intervention), considering two factors: training protocol (one, two, or three sets) and time of assessment. For this purpose, a repeated‐measures analysis of variance was performed, with Greenhouse–Geisser correction applied when the sphericity assumption was not met. For pain intensity, repeated‐measures ANOVA was used to evaluate the effects of training protocol, assessment time, and their interaction. When appropriate, Greenhouse–Geisser correction was applied, followed by Bonferroni‐adjusted post hoc comparisons.

Variables related to training load and the number of repetitions obtained during submaximal repetition testing were described using means and standard deviations for loads, and medians with minimum and maximum values for repetitions. No inferential analyses were performed for these variables, as they were descriptive of the load prescription procedure.

Exercise execution time was compared across training protocols using repeated‐measures analysis of variance, with training protocol treated as a within‐subject factor. When necessary, the Greenhouse–Geisser correction was applied. For all inferential analyses, a significance level of 5% was adopted (*p* < 0.05).

In addition to null hypothesis significance testing, effect sizes were calculated to quantify the magnitude of statistically significant between‐group differences and to complement the interpretation of *p* values. For comparisons involving continuous outcomes, Cohen's *d* was considered the appropriate measure of effect size, whereas Cohen's *w* was used for analyses based on the chi‐square test of proportions. According to conventional thresholds, Cohen's *d* values of 0.2, 0.5, and 0.8 represent small, moderate, and large effects (J. Cohen 1988), respectively, while Cohen's *w* values of 0.1, 0.3, and 0.5 indicate small, moderate, and large effects, respectively. To avoid overinterpretation of nonsignificant findings, effect sizes were reported only for statistically significant between‐group comparisons (Pontes‐Silva 2023).

## 3 Results

Of the 248 participants initially screened for eligibility, 38 met the inclusion criteria and were randomized after the baseline assessment. All participants completed the intervention protocols and scheduled assessments, with no withdrawals at any assessment time point. Accordingly, the study achieved a 100% adherence rate, with no sample loss during the intervention period. The flow of participants throughout the study is presented in Figure 1.

**FIGURE 1:**
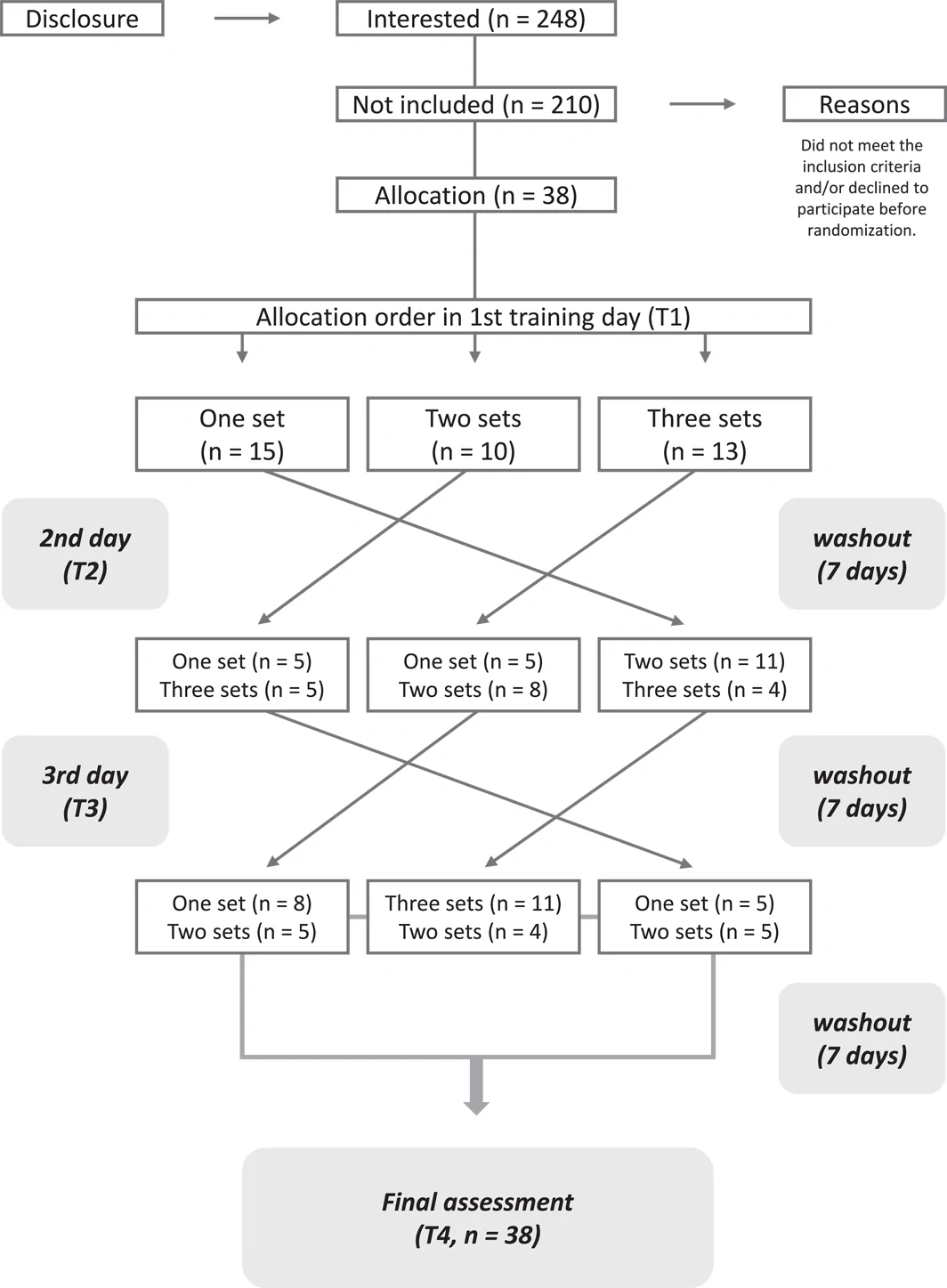
Flowchart.

The characteristics of the sample are presented in Table 3. Participants had a mean age of 41.2 ± 9.7 years and a mean duration of fibromyalgia diagnosis of 7.1 ± 5.3 years. Fibromyalgia impact, assessed using the Fibromyalgia Impact Questionnaire–Revised (FIQR), indicated a moderate level of impairment, with a mean total score of 66.1 ± 17.1 points. Regarding physical activity and sedentary behavior, substantial variability was observed in estimated weekly energy expenditure from exercise, along with an average sitting time of approximately 6 hours per day.

**TABLE 3:** Baseline anthropometric, clinical, and behavioral characteristics of the participants (*n* = 38).

Variable	Mean ± SD [Min–Max]	95% CI
Age (years)	41.2 ± 9.7 [23.0–59.0]	38–44
Time since fibromyalgia diagnosis (years)	7.1 ± 5.3 [1.0–20.0]	5.4–8.9
Body mass (kg)	70.7 ± 13.9 [50.0–109.0]	66–75
Height (m)	1.60 ± 0.10 [1.50–1.80]	1.60–1.60
Fibromyalgia impact questionnaire–Revised		
Physical function	16.5 ± 7.0 [2.0–30.0]	14–19
Global impact	14.0 ± 5.8 [0.0–20.0]	12–16
Symptoms	35.6 ± 8.8 [17.0–50.0]	33–38
Total score	66.1 ± 17.1 [32.0–96.0]	61–72
Estimated weekly exercise energy expenditure (MET·min/week)	8921.7 ± 13,875.2 [0.0–74,340.0]	4361–13,482
Estimated weekly exercise energy expenditure (kcal/week)	10,691.1 ± 15,096.1 [0.0–65,047.5]	5729–15,653
Total sitting time per week (hours)	41.7 ± 20.9 [9.0–98.0]	35–49
Average daily sitting time (hours/day)	6.0 ± 3.0 [1.3–14.0]	5.0–6.9

*Note:* Values are presented as mean ± standard deviation (SD) [minimum–maximum].

Abbreviation: CI, confidence interval.

The distribution of sociodemographic characteristics, lifestyle habits, and continuous medication use is shown in Table 4. Most participants were non‐smokers (95%), with a higher prevalence of women who were married or single, self‐identified as Brown or White, and had completed at least a secondary level of education. The majority of participants reported continuous medication use (71%). Statistically significant differences were observed in the distribution of smoking status, marital status, ethnicity, educational level, and medication use (*p* < 0.05), whereas income distribution did not differ significantly. Most participants were classified as active or very active (87%), while only one participant was classified as sedentary. The distribution of physical activity categories differed significantly from the expected proportions (*p* < 0.001).

**TABLE 4:** Sociodemographic characteristics, lifestyle factors, and medication use of the participants (*n* = 38).

Variable	*n* (%)	95% CI	*χ* ^2^ (*p* value)
Smoking status			
Non‐smoker	36 (95.0)	81–99	30.421 (< 0.001)
Smoker	2 (5.3)	0.92–19	
Marital status			
Single	12 (31.6)	18–49	19.632 (0.001)
Married	15 (39.5)	24–57	
Widowed	2 (5.3)	0.92–19	
Divorced	8 (21.1)	10–38	
Prefer not to answer	1 (2.6)	0.14–15	
Ethnicity			
White	14 (36.8)	22–54	22.000 (< 0.001)
Asian	3 (7.9)	2.1–22	
Brown	19 (50.0)	35–65	
Indigenous	2 (5.3)	0.92–19	
Monthly household income			
≤ 1 minimum wage	6 (15.8)	6.6–32	6.421 (0.098)
2–3 minimum wages	16 (42.1)	27–59	
≥ 4 minimum wages	9 (23.7)	12–41	
Prefer not to answer	7 (18.4)	8.3–35	
Physical activity level (IPAQ)			
Very active	9 (23.7)	12–41	50.684 (< 0.001)
Active	24 (63.2)	46–78	
Irregularly active A	4 (10.5)	3.4–26	
Irregularly active B	0 (0.0)	0.0–11	
Sedentary	1 (2.6)	0.14–15	
Educational level			
Primary education	3 (7.9)	2.1–22	12.053 (0.002)
Secondary education	15 (39.5)	24–57	
Higher education	20 (52.6)	36–69	
Continuous medication use			
No	11 (28.9)	16–46	6.737 (0.014)
Yes	27 (71.1)	54–84	

*Note:* Values are presented as *n* (%). χ^2^: chi‐square test. Minimum wage refers to the national minimum wage at the time of data collection.

Abbreviation: CI, confidence interval.

This distribution indicates that the study sample was composed predominantly of physically active women, despite the exclusion of individuals who had participated in resistance training during the previous 6 months. Therefore, the IPAQ classification should be interpreted as reflecting habitual physical activity rather than a recent engagement in structured resistance exercise.

Participants' preferences regarding resistance training volume are presented in Table 5. No participant expressed a preference for the one‐set protocol, whereas the two‐ and three‐set protocols were equally preferred, each selected by 50% of the sample. The distribution of preferences differed significantly across training volumes (*p* < 0.001). The corresponding effect size for the chi‐square analysis was large (Cohen's *w* = 0.71), indicating a substantial difference in participants' preferences across the three training volumes, primarily driven by the complete absence of preference for the one‐set protocol.

**TABLE 5:** Distribution of participant preferences for resistance training volume (*n* = 38).

Preferred training volume	*n* (%)	95% CI
One set	0	0.0–11
Two sets	19 (50.0)	35–65
Three sets	19 (50.0)	35–65

*Note:* Chi‐square test: *p* < 0.001.

Treatment expectancy, assessed prior to the execution of the training protocols, did not differ significantly among the three training volumes (Table 6). Mean expectancy scores were comparable across the one‐, two‐, and three‐set protocols (*p* = 0.20). Pain intensity was assessed at four time points (baseline, pre‐exercise, post‐exercise, and 24 hours post‐intervention) for each training protocol, as shown in Table 2. No significant differences were observed between the protocols at any assessment time point. Within‐protocol analyses revealed a significant effect of time only for the two‐set protocol (*p* = 0.015). Post hoc pairwise comparisons demonstrated significantly lower pain intensity at post‐exercise compared with both baseline and pre‐exercise assessments, whereas no significant differences were observed between post‐exercise and 24 hours post‐exercise or among any other time‐point comparisons. The one‐ and three‐set protocols did not show statistically significant temporal changes.

**TABLE 6:** Treatment expectancy across resistance training volumes (*n* = 38).

Training volume	Treatment expectancy (mean ± SD)
One set	3.63 ± 0.94
Two sets	3.97 ± 0.82
Three sets	3.95 ± 1.23

*Note:* Between‐group comparison: One‐way ANOVA, *p* = 0.20.

Considering the predefined minimal clinically important difference of *a* ≥ 2‐point reduction on the Numeric Pain Rating Scale, the mean changes observed across all training volumes did not reach this threshold at any assessment time point. Although the two‐set protocol demonstrated a statistically significant reduction in pain over time, the magnitude of change remained below the established criterion for clinical importance, indicating that the observed effect should be interpreted as statistically significant but not clinically meaningful.

Training loads and the number of repetitions used for exercise prescription are described in Table 7. Mean loads varied across exercises and were individually determined based on submaximal repetition testing, while the number of repetitions performed during the protocols was standardized at 10 repetitions for all exercises and training volumes. Exercise execution time across the different training protocols is presented in Table 8. No statistically significant differences in execution time were observed between the one‐, two‐, and three‐set protocols for any of the exercises analyzed (*p* > 0.05).

**TABLE 7:** Training loads and repetitions obtained from submaximal repetition testing and execution during resistance training sessions (*n* = 38).

Exercise	Testing phase	Repetitions, median (min–max)	Execution phase	Repetitions
Variable	Load (kg) mean ± SD		Load (kg), mean ± SD	
Seated leg curl	24.3 ± 4.6	15.0 (9–32)	20.4 ± 4.4	10
Leg press	10.4 ± 8.5	13.5 (8–30)	8.6 ± 7.2	10
Front lat pulldown	18.0 ± 3.4	14.5 (6–25)	14.7 ± 3.0	10
Bench press	3.8 ± 2.7	11.5 (6–25)	3.0 ± 2.1	10

*Note:* The loads reported in this table correspond exclusively to the additional weight plates (external load) added to each resistance machine and do not include the intrinsic mechanical resistance of the equipment. Consequently, these values should not be interpreted as the total effective load lifted by participants.

**TABLE 8:** Exercise execution time across resistance training volumes (*n* = 38).

Exercise	One set(s) mean ± SD	Two sets(s) mean ± SD	Three sets(s) mean ± SD	*p* value
Seated leg curl	28.05 ± 8.33	28.36 ± 9.09	27.60 ± 8.66	0.711†
Leg press	29.26 ± 8.09	27.86 ± 8.32	28.34 ± 8.80	0.292†
Front lat pulldown	29.18 ± 7.55	30.00 ± 7.14	29.63 ± 6.14	0.532
Bench press	28.21 ± 8.03	26.88 ± 7.52	27.04 ± 6.82	0.181

^†^ *p* values were adjusted using the Greenhouse–Geisser correction when the sphericity assumption was violated.

## 4 Discussion

The present study investigated participants' preferences and acute subjective responses to different resistance training volumes in women with fibromyalgia, adopting a randomized crossover design centered on participant experience rather than long‐term clinical efficacy. The main finding was that no participant identified the one‐set protocol as her preferred training volume, whereas the two‐ and three‐set protocols were equally preferred. This result highlights the relevance of training volume as a determinant of participant preference and supports the integration of patient preference into resistance exercise prescription for fibromyalgia.

The finding that no participant selected the one‐set protocol as her preferred option is noteworthy, as lower exercise volumes are often presumed to be more suitable for individuals with chronic pain conditions. However, this assumption may overlook the importance of perceived adequacy and engagement. One possible explanation is that lower training volumes may have been perceived by some participants as providing a less engaging or less meaningful exercise experience. However, the present study did not directly assess the reasons underlying participants' preferences, and this interpretation should therefore be considered speculative. This interpretation aligns with evidence suggesting that exercise adherence is influenced not only by symptom response but also by perceived effectiveness and personal relevance of the intervention (Lumley et al. 2011).

The equal preference observed between the two‐ and three‐set protocols suggests that moderate to higher volumes are both acceptable within an acute training context. Importantly, treatment expectancy did not differ between protocols, indicating that preference was unlikely to be driven by anticipatory beliefs. This finding strengthens the internal validity of the preference outcome and supports the interpretation that experiential factors during and after exercise played a central role.

An important characteristic of the present sample is that most participants were classified as physically active according to the IPAQ. Although none had engaged in resistance training during the previous 6 months, this relatively active profile may have influenced their acceptance of moderate and higher training volumes. Consequently, the observed preferences may not be fully generalizable to more sedentary populations with fibromyalgia, who frequently exhibit lower levels of habitual physical activity and greater exercise intolerance.

Regarding pain intensity, a significant effect of time was observed only in the two‐set protocol. Post hoc analyses indicated that pain intensity was significantly lower immediately after exercise compared with both baseline and pre‐exercise assessments, whereas no significant differences were observed for the remaining comparisons or in the one‐ and three‐set protocols.

Although no between‐protocol differences were detected, this within‐protocol effect may reflect a favorable balance between mechanical stimulus and tolerability. Moderate resistance training volumes have been proposed as sufficient to activate analgesic mechanisms, such as endogenous pain modulation, without exceeding individual tolerance thresholds (Edwards et al. 2017). Nevertheless, given the exploratory nature of this analysis and the lack of a priori hypotheses regarding analgesic superiority, this finding should be interpreted cautiously and not as evidence of clinical effectiveness.

Furthermore, despite statistical significance within the two‐set protocol, the average reduction did not achieve the predefined minimal clinically important difference of two points on the Numeric Pain Rating Scale. This distinction between statistical significance and clinical relevance is particularly important in pain research, where small numerical changes may not translate into perceptible benefits for patients (Farrar et al. 2001).

The similarity in exercise execution time, subjective effort, and prescribed loads across protocols further supports the conclusion that training volume was the primary distinguishing factor influencing preference. This is clinically relevant, as it suggests that differences in preference were not attributable to disparities in intensity, effort, or pacing, but rather to the perceived adequacy of the training dose. From a rehabilitation perspective, this reinforces the conceptualization of training volume as analogous to medication dosage, where both underdosing and overdosing may negatively affect patient engagement (Rausch Osthoff et al. 2018).

Patient‐centered care has been consistently recommended in fibromyalgia management, particularly in the context of exercise prescription. Clinical guidelines emphasize the importance of tailoring interventions to individual capabilities, symptoms, and preferences to enhance long‐term adherence (Wolfe et al. 2016). The present findings contribute to this body of evidence by demonstrating that, in this sample, no participant selected the one‐set protocol as the preferred option, whereas the two‐ and three‐set protocols were equally preferred. These findings suggest that very low training volumes may not necessarily be preferred by women with fibromyalgia, although confirmation in other populations is warranted.

Several limitations should be acknowledged. The study evaluated acute responses and preferences, which may differ from long‐term perceptions and adherence patterns. Additionally, the sample size, although adequate for the primary outcome, limits the generalizability of secondary findings. Another limitation is that the study population was relatively young and predominantly classified as active or very active according to the IPAQ. This profile may limit the generalizability of the findings to a broader population with fibromyalgia, particularly older and less physically active individuals. The relatively young age of the sample reflects the prespecified eligibility criterion of 18–65 years, which was adopted to reduce potential heterogeneity related to age‐associated comorbidities. The high level of habitual physical activity may also have influenced participants' perceptions of and preferences for resistance training volume. Therefore, replication of these findings in older and less physically active cohorts is warranted. Although the intervention order was randomized, potential order or recency effects cannot be completely excluded, as participants' preferences may have been influenced by experiences from previous sessions.

In summary, this study provides novel evidence that women with fibromyalgia preferentially selected resistance training protocols involving two or three sets, whereas none identified the one‐set protocol as their preferred option. These findings underscore the importance of incorporating patient preference into resistance training prescriptions and support a shift toward more individualized, experience‐informed exercise strategies in fibromyalgia care.

In conclusion, women with fibromyalgia demonstrated equivalent preference for resistance training protocols involving two and three sets, with each protocol being selected by 50% of the participants, whereas no participant identified the one‐set protocol as the preferred option. Treatment expectancy did not differ among the training volumes, and no clinically meaningful differences in pain intensity were observed following the interventions. These findings highlight the importance of considering patient preference when prescribing resistance training and support the use of individualized exercise strategies in the management of fibromyalgia. Future studies should investigate whether these preferences are maintained over longer intervention periods and whether they are associated with improved adherence and clinical outcomes.

## 5 Implications of Physiotherapy Practice

— Resistance training programs for fibromyalgia should consider patient preference to support individualized exercise prescription and acceptability.
— Two‐ and three‐set protocols may both be considered viable options, as they were equally preferred by participants and no clinically meaningful differences in pain were observed between protocols.
— Physiotherapists should incorporate subjective experience alongside pain outcomes when prescribing resistance exercise.

## Author Contributions

M.C.d.S. and B.H.C.: conceptualization. B.H.C. and G.M.R.: data curation, investigation, and statistical analysis. All authors: methodology, validation, visualization, writing (original draft, review, and editing).

## Funding

The authors have nothing to report.

## Ethics Statement

This study was approved by the Research Ethics Committee of the Universidade Federal do Rio Grande do Norte (report number: 6.303.419; October 20, 2024).

## Consent

Informed consent was obtained from all subjects and/or their legal guardian(s). All respondents participated in this study freely and with their consent. All experiments were conducted in accordance with the tenets of the Declaration of Helsinki.

## Conflicts of Interest

Dr. André Pontes‐Silva serves as a reviewer for the *Physiotherapy Research International*. The authors declare no further competing interests.

## Data Availability

The data that support the findings of this study are available on request from the corresponding author. The data are not publicly available due to privacy or ethical restrictions.
